# Job Burnout Is Associated With Prehospital Decision Delay: An Internet-Based Survey in China

**DOI:** 10.3389/fpsyg.2022.762406

**Published:** 2022-04-11

**Authors:** Han Yin, Cheng Jiang, Xiaohe Shi, Yilin Chen, Xueju Yu, Yu Wang, Weiya Li, Huan Ma, Qingshan Geng

**Affiliations:** ^1^Department of Cardiology, Guangdong Cardiovascular Institute, Guangdong Provincial People's Hospital, Guangdong Academy of Medical Sciences, Guangzhou, China; ^2^School of Medicine, South China University of Technology, Guangzhou, China; ^3^Department of Cardiac Rehabilitation, Guangdong Cardiovascular Institute, Guangdong Provincial People's Hospital, Guangdong Academy of Medical Sciences, Guangzhou, China

**Keywords:** burnout, time-to-treatment, social support, type D personality, decision making

## Abstract

**Background:**

Prehospital delay is associated with non-modifiable factors such as age, residential region, and disease severity. However, the impact of psychosocial factors especially for job burnout on prehospital decision delay is still little understood.

**Method:**

This internet-based survey was conducted between 14 February 2021 and 5 March 2021 in China through the Wechat platform and web page. Self-designed questionnaires about the expected and actual length of prehospital decision time and the Chinese version of Maslach Burnout Inventory-General Survey, Type D Personality Scale-14, and Social Support Rating Scale were applied. A total of 1,039 general participants with a history of perceptible but tolerable body discomfort were included.

**Results:**

The top six reasons for prehospital decision delay were: (1) endure until self-healing (50.7%), (2) too busy to ask for leave (40.3%), (3) process for seeing a doctor too complicated (35.8%), (4) too tired after work (26.2%), (5) worry about the expenditure (16.6%), and (6) fear of being identified as with serious problem (14.5%). The univariate analyses revealed that older age (*p* = 0.001), type D personality (*p* = 0.025), job burnout (*p* = 0.055), and worrying about expenditure (*p* = 0.004) were associated with prolonged prehospital decision time, while engaged in medical-related job (*p* = 0.028) and with more social support (*p* = 0.066) would shorten the delay. The multivariate analysis using logistic regression model with forward selecting method showed that age [per 10 years, odds ratio (OR) 1.19 (1.09–1.31), *p* < 0.001], job burnout [per 10 points in Maslach Burnout Inventory-General Survey (MBI-GS), OR 1.17 (1.04–1.31), *p* = 0.007], and worrying about expenditure [OR 1.75 (1.25–2.47), *p* = 0.001] were the three determinants for prehospital decision delay (>7 days). Mediating effects were analyzed by using bias-corrected percentile bootstrap methods (*N* = 10,000). Social support was found partially mediated the relationship between the determinants and prehospital decision time. The partial mediating effect of social support accounted for 24.0% of the total effect for job burnout and 11.6% for worrying about expenditure.

**Conclusion:**

Psychosocial factors have a non-negligible impact on prehospital decision delay. The crucial part of prehospital decision delay may be the lack of motivation inside. Job burnout and lack of social support, as two commonly seen features in the modern world, should be given enough consideration in disease prevention and treatment.

## Introduction

Delay in seeking medical help can profoundly increase the risk for serious complications, major disability, and death (De Luca et al., 2004; Denti et al., 2016; van Dijk et al., 2018). Previous studies have focused on diseases such as acute myocardial infarction (AMI) and stroke (De Luca et al., 2004; Denti et al., 2016) and found that lack of disease-related knowledge, older age, and residing in rural areas were associated with prehospital delay (Cao et al., 2010; Mohan et al., 2018; Park et al., 2020). However, these factors are often non-modifiable and likely account for only a small proportion of prehospital delay (George et al., 2017). Besides, the behavior of seeking medical aids for such acute diseases was to a large extent driven by symptoms (Wu et al., 2017). For diseases at the initial stage without intense manifestations such as cancers, the prehospital delay may cause more mournful outcomes.

Psychosocial factors such as depression, type D personality, and lack of social support have been linked to a worse prognosis of several somatic diseases (Hare et al., 2014) and reported in one recent article to be associated with prehospital decision delay in patients with AMI (Arrebola-Moreno et al., 2020b). It was also mentioned in another research that patients' negative trustworthiness of others was independently associated with an increased intention to wait before going to the hospital (Sullivan et al., 2009). Given the fact that a large part of the delay time for seeking medical care is due to the patient's decision delay instead of some objective reasons (Mackay et al., 2014), it is possible that psychosocial problems and their related behaviors may be a pivotal factor in causing prehospital delay (Bunde and Martin, 2006; Smolderen et al., 2010). More importantly, these problems are often neglected but the modifiable parts for the individual in clinical practice and exert significant influence on prognosis through affecting the decision-making process (Arrebola-Moreno et al., 2020b; Veazie and Denham, 2021) and medical compliance (Acharya and Agius, 2018).

Characterized by feeling exhaustion, cynicism, and reduced personal accomplishment, job burnout has been considered to affect both the physical and psychological states (Maslach and Leiter, 2016) and is associated with an increased risk of somatic diseases and their progression (von Känel et al., 2020). It depletes energy and makes employees more likely to delay decisions (Roster and Ferrari, 2020) and trapped into a risky decision-making style (Michailidis and Banks, 2016). Considering the necessity of work in our life and also the increasing social competition, it is worthwhile to figure out the impact of job burnout on prehospital decision delay. To the best of our knowledge, this relationship has not been explored before.

As for these reasons above, we initiated this research in the general population and focused on the influence of psychosocial factors especially for job burnout on the delay before seeking medical help in individuals previously having perceptible but tolerable body discomfort. We hypothesized that (1) job burnout would postpone the prehospital decision time and (2) social support might mediate the relationship between burnout and prehospital decision delay. We expected that these modifiable factors could be associated with the delay in seeking medical care so that provide new insights for improving the current disease prevention system.

## Methods

### Design and Participants

This study aimed to explore the influence of psychological factors, especially for job burnout on the delay in seeking medical help in individuals previously having perceptible but tolerable body discomfort. It was conducted based on the Internet through the Wechat platform and web page (available on https://www.wjx.cn/vj/r4hD79V.aspx) between 14 February 2021 and 5 March 2021. Participants with a history of perceptible tolerable body discomfort and a history of visiting doctors were eligible for this research. All the participants needed to check their qualifications and to read and tick the box of informed consent on the web page before enrollment. In the survey, they were asked to recall the experiences of visiting hospitals and to calculate the actual length from the beginning of body discomfort to visiting doctors. Self-designed questionnaire (see Supplementary File 1) about demographic characteristics, expected length of prehospital decision time, reasons for prehospital decision delay, and modified form of Maslach Burnout Inventory-General Survey (MBI-GS), Type D Personality Scale-14 (DS14), and Social Support Rating Scale (SSRS) were also included in this survey.

To avoid data missing, all the questionnaires were mandatory and the web page would remind the participants if there was an item blanked when submitting. In all, 1,039 participants across China from 29 provinces or autonomous regions attended this survey. However, questionnaires that were completed in <100 s were ruled out in consideration for quality control, leaving a sample size of 1,032 into analysis. Among those participants, 400 (38.8%) were men, 380(36.8%) were aged <30 years, and 229 (22.2%) were engaged in medical-related jobs. This research complied with the Declaration of Helsinki and was approved by the ethics committee.

### Expected Length of Prehospital Decision Time

Participants were asked to imagine a scenario that some perceptible but tolerable body discomfort had for the first time occurred on them. The ideal length of prehospital decision time in expectation based on their cognition was inquired.

### Modified Form of Maslach Burnout Inventory-General Survey (Chinese Version)

As a widely used questionnaire, MBI-GS consists of 16 items (0–6 Likert-type scale: 0 = never, 6 = everyday), 3 dimensions (emotional exhaustion, cynicism, and reduced personal accomplishment), with higher scores indicating higher levels of job burnout (Leiter and Robichaud, 1997). The modified form of MBI-GS (Chinese version) deletes the 13th item about cynicism, but keeps the same structure and good reliability and validity (Gan et al., 2019). A total score of 51–75 indicates mild job burnout, whereas a total score >75 represents moderate to severe burnout, for which leaving the post for a period of time for adjustment is suggested.

### Type D Personality Scale-14 (Chinese Version)

Type D personality was assessed by the Chinese version of the Type D Personality Scale-14. It comprises two 7-item subscales to measure negative affectivity (NA) (e.g., “I often feel unhappy”) and social inhibition (SI) (e.g., “I am a closed kind of person”) with a maximum score of 28 on each scale. Type D personality was classified as scores of both two subscales ≥10 (Pedersen et al., 2004). The inner and retest consistency and content and structure validity have been validated in the general Chinese population (Bai and Zhao, 2007).

### Social Support Rating Scale

Social Support Rating Scale was designed in accordance with the actual living state of Chinese people by referring other foreign scales (Chang-Fei et al., 2011). It consists of 14 items and assesses the level of social support from 3 dimensions of objective support, subjective support, and utilization of support. Its reliability and validity have been validated in general population. More details about this questionnaire were attached in Supplementary File 1. A good internal consistency (Cronbach's α = 0.79) was observed. As there has not been a widely recognized cutoff point for the scale and higher score indicates a higher level of social support, we adopted the lower quartile of 30 points for categorization in the dichotomous analysis.

### Statistical Analyses

The actual length of prehospital decision time of participants in different demographic and psychosocial categorizations was presented as means (SDs) and compared between/among groups with the Wilcoxon rank-sum test or the Kruskal–Wallis test due to the skew distribution. Prehospital decision delay was defined as actual prehospital decision time exceeding 7 days. The multivariate logistic regression model with forward selection method (sle = 0.1, sls = 0.1) was applied to find out the main determinants for prehospital decision delay by including all statistically significant variables in the univariate analyses. The correlations between variables were assessed by the Spearman correlation coefficient. These analyses were performed with SAS version 9.4 (SAS Institute Incorporation, Cary, North Carolina, USA). The mediating effects of social support, type D personality, and medical-related jobs in the relationship between determinants and prehospital decision time were examined with bias-corrected percentile Bootstrap methods by Mplus version 8.0.

Based on our pilot data, about 40% of the general population in China would have job fatigue. The rate of prehospital decision delay was estimated at 50% in individuals with at least mild burnout and 41% in those without job fatigue. With at least 80% power, the sample size was calculated to be approximately 1,000 by using PASS 15 software. The actual prevalence gap was slightly lower than expected, causing the power of this research to be at 74.4%. All the tests for significance were two-tailed at the threshold of 0.05.

## Results

### Characteristics of Participants

**Figure 1:**
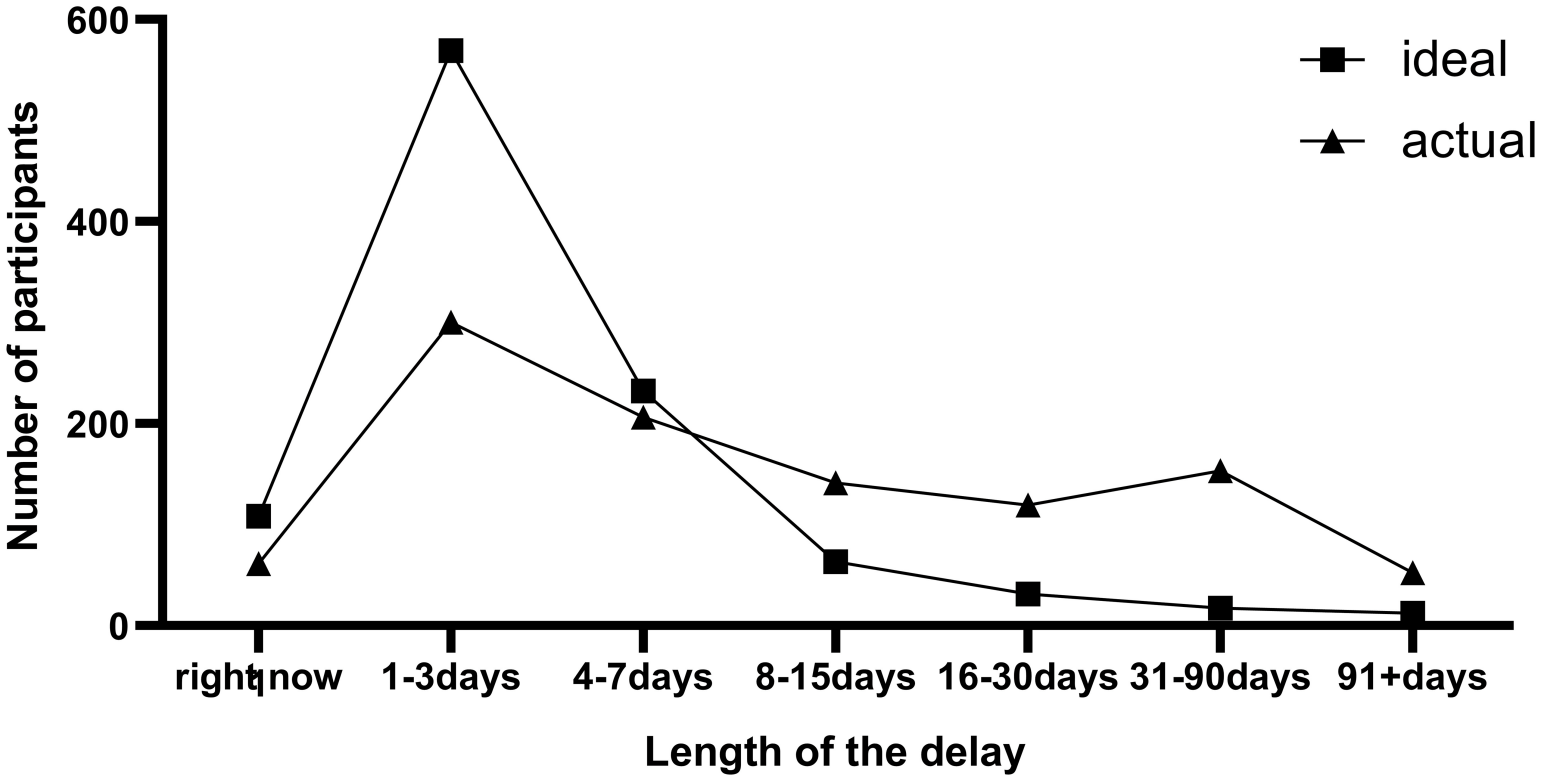
Frequency distribution of the expected and actual delay for seeking medical help (*N* = 1,032).

The demographic and psychological characteristics of the participants are shown in Table 1. Of the 1,032 subjects, 311 (30.1%) had type D personality, 431 (41.8%) had at least mild job burnout, and 13(1.3%) were in the state of moderate to severe burnout. Although nearly half of participants (45.1%) actually sought medical help after 1 week, one fifth (19.9%) after 1 month since the first time that some unexpected, perceptible but tolerable body discomfort occurred on them, the majority (88.1%) believed that the ideal decision delay should be within 1 week (Figure 1). For this reason, we defined that a prehospital decision time exceeding 7 days could be regarded as prehospital decision delay. Consequently, the prevalence of prehospital decision delay was 48.5% in participants with job burnout and 42.6% in their counterparts.

**Table 1:** Summary of the associations between demographic, psychological characteristics, and prehospital decision time (*n* = 1,032).

**Variable**	**Categorization**	***N***	**Length of delay in seeking medical help [days, mean (SD)]**	***p*-value**
Gender	Male	400	21.97 (37.68)	0.44
	Female	632	19.83 (33.99)	
**Age**	10–19	21	32.67 (62.22)	**0.001**
	20–29	359	17.35 (32.57)	
	30–39	194	16.98 (29.25)	
	40–49	169	21.77 (33.99)	
	50–59	239	26.81 (41.90)	
	60–	50	20.50 (29.80)	
Income	0–3,000	309	21.44 (36.09)	0.54
	3,000–6,000	273	22.49 (37.88)	
	6,000–10,000	250	17.56 (29.45)	
	10,000–20,000	153	19.11 (35.48)	
	20,000–	47	26.51 (44.82)	
**Medical related job**	Yes	229	15.97 (29.28)	**0.028**
	No	803	22.00 (36.95)	
Physical examination frequency	Almost never	205	26.57 (43.47)	0.11
	<1 time per year	288	21.64 (35.28)	
	≥ 1 times per year	539	17.89 (31.75)	
**Type D personality**	Yes	311	24.06 (40.10)	**0.025**
	No	721	19.19 (33.19)	
Burnout	<50	601	19.05 (33.74)	0.055
(MBI-GS)	50–75	418	22.36 (36.50)	
	≥75	13	40.38 (64.80)	
Social support	≤30	280	26.49 (44.09)	0.066
(SSRS)	>30	752	18.49 (31.42)	
**Seeking medical help in time**	Always	683	13.16 (23.76)	**<0.001**
	Sometimes	203	26.23 (33.28)	
	Seldom	146	48.01 (60.68)	
**Reasons**			
1. Hold on a little longer, might be OK	With	523	19.33 (33.65)	0.67
	Without	509	22.02 (37.22)	
2. Too busy at workdays, hard to find time	With	416	19.22 (34.01)	0.39
	Without	616	21.62 (36.39)	
3. Process for seeing a doctor too complicated	With	369	19.27 (33.02)	0.62
	Without	663	21.44 (36.75)	
4. Too tired after work, prefer to rest	With	270	21.37 (36,74)	0.37
	Without	762	20.41 (35.03)	
**5. Worried about the expenditure**	With	171	27.66 (44.16)	**0.004**
	Without	861	19.27 (33.32)	
6. Fear of being identified as with serious problem	With	150	23.43 (36.63)	0.11
	Without	882	20.19 (35.26)	

*The length of prehospital decision time was presented as means (SDs), however, were compared with Wilcoxon rank-sum test or Kruskal–Wallis test. MBI-GS, Maslach Burnout Inventory-General Survey; SSRS, Social Support Rating Scale. Variables marked in bold indicate a statistically significant differece between/among groups*.

In addition, we also investigated the first choice when facing that situation and noticed that 405 (39.2%) chose to endure for a while to see if the symptoms could disappear, 262 (25.4%) would search the symptoms on Internet, 99 (9.6%) tried to consult their relatives or friends in the medical field, and only 197 (19.1%) would immediately access to the nearby clinics or hospitals.

### Associations Between Prehospital Decision Delay and Characteristics of Participants

In the univariate analyses, older age (*p* = 0.001), type D personality (*p* = 0.025), and job burnout (*p* = 0.055) were associated with prolonged prehospital decision time, while engaged in medical-related job (*p* = 0.028) and with more social support (*p* = 0.066) would shorten the delay in seeking medical help.

**Figure 2:**
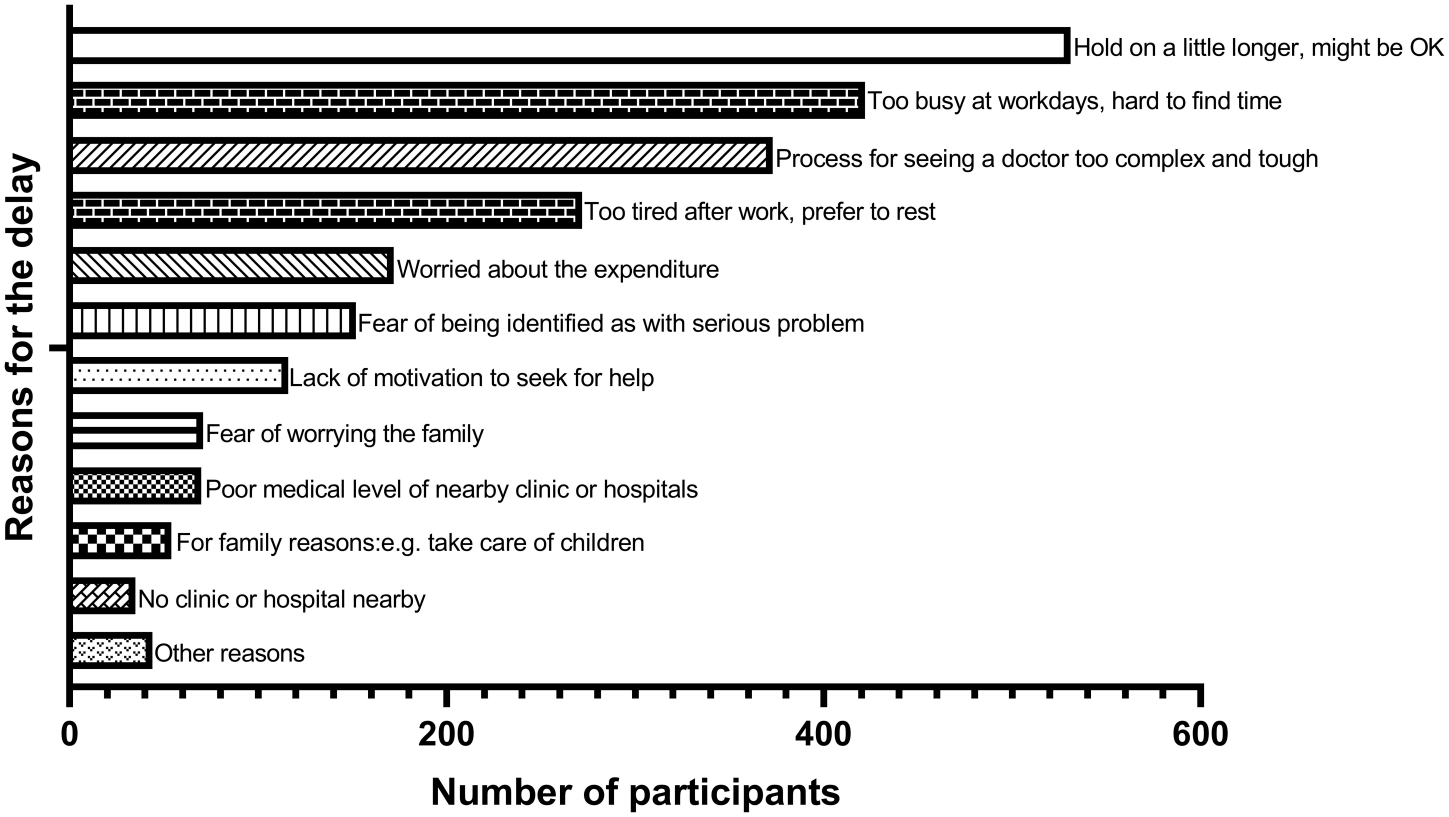
Ranking for reasons of the delay for seeking medical help (*N* = 1,032).

Reasons for prehospital decision delay had been collected in the pilot study and later been placed in our questionnaire. The top six reasons (Figure 2) for the delay were (1) personal reason: endure until self-healing (50.7%); (2) job reason: too busy to ask for leave (40.3%); (3) objective reason: process for seeing a doctor too complicated and troublesome (35.8%); (4) job reason: too tired after work and prefer to rest (26.2%); (5) economic reasons: worry about the expenditure (16.6%); and (6) personal reason: fear of being identified as with serious problem (14.5%). To our surprise, none of these reasons, neither the level of income, but for having worries about the possible expenditure (*p* = 0.004) were in fact associated with delayed decision time (see Table 1). Those who felt afraid of the possible bad results were prone to have a longer mean prehospital decision time (*p* = 0.11). People who felt busy at work, preferred to endure discomfort, or felt the process to see the doctor too complicated even had a slightly shorter mean prehospital decision time.

Multivariate analysis taking all these factors into logistic regression model with forward selection method (sle = 0.1, sls = 0.1) revealed that job burnout [every 10 points increase in MBI-GS, odds ratio (OR) 1.17 (1.04–1.31), *p* = 0.007], age [every 10 years increase, OR 1.19 (1.09–1.31), *p* < 0.001], and worrying about expenditure [OR 1.75 (1.25–2.47), *p* = 0.001] were the three determinants for the delay in seeking medical help.

### Job Burnout, Age, Social Support, and Prehospital Decision Delay

To fully understand the inner relationships between all these variables, Spearman's rank correlation tests were further conducted (see Table 2). Intercorrelates among variables were in line with our expectations. Job burnout (*r* = 0.11, *p* < 0.001), age (*r* = 0.13, *p* < 0.001), and worrying about expenditure (*r* = 0.09, *p* = 0.004) correlated positively, while SSRS score (*r* = −0.05, *p* = 0.081) and be occupied in medical-related job (*r* = −0.07, *p* = 0.028) correlated negatively with prehospital decision time. Type D personality (*r* = 0.07, *p* = 0.024) was another predominant associated factor, though the correlation as not as strong as negative affectivity (*r* = 0.11, *p* < 0.001).

**Table 2:** Spearman's correlations between prehospital decision time and demographic, psychological features (*N* = 1,032).

	**1**	**2**	**3**	**4**	**5**	**(NA)**	**(SI)**	**6**	**(EE)**	**C**	**(RPA)**	**7**
1 Prehospital decision time	–											
2 Age	0.13^***^	–										
3 Education level	−0.08^**^	−0.42^***^	–									
4 Medical related job	−0.07^*^	−0.30^***^	0.36^***^	–								
5 Type D personality	0.07^*^	−0.12^***^	0.04	0.02	–							
Negative affectivity (NA)	0.11^***^	−0.11^***^	0.04	0.01	0.63^***^	–						
Social inhibition (SI)	0.08^**^	−0.12^***^	0.06^*^	0.01	0.74^***^	0.62^***^	–					
6 Job burnout (MBI-GS)	0.11^***^	−0.19^***^	0.04	0.04	0.40^***^	0.52^***^	0.41^***^	–				
Emotional exhaustion	0.06	−0.24^***^	0.22^***^	0.13^***^	0.31^***^	0.45^***^	0.34^***^	0.69^***^	–			
Cynicism	0.08^*^	−0.22^***^	0.17^***^	0.07^*^	0.40^***^	0.53^***^	0.41^***^	0.78^***^	0.70^***^	–		
Reduced personal accomplishment	0.10^**^	0.05	−0.24^***^	−0.09^**^	0.15^***^	0.13^***^	0.14^***^	0.52^***^	−0.12^***^	0.04	–	
7 Social support (SSRS)	−0.05	0.34^***^	−0.17^***^	−0.11^***^	−0.34^***^	−0.36^***^	−0.42^***^	−0.32^***^	−0.23^***^	−0.28^***^	−0.16^***^	−

* *p < 0.05*,

** *p < 0.01, and*

*** *p < 0.001*.

*MBI-GS, Maslach Burnout Inventory-General Survey; SSRS, Social Support Rating Scale*.

Due to the existence of collinearity between variables, the interrelationship of some factors might be overlooked. Therefore, we tried to testify the mediating effect of social support, type D personality, and medical-related jobs between each of the three determinants (job burnout, age, and worrying about expenditure) with prehospital decision time by using bias-corrected percentile Bootstrap methods (iteration = 10,000 times). These results are given in Table 3. Obviously, social support partially mediated the relationship between job burnout and decision time and the relationship between worrying about expenditure and decision time, which suggested that the length of the time before seeking medical help might be shortened by strengthening social support for individuals. It however diluted the relationship between age and prehospital decision time, indicating that the influence of aging on prehospital delay could be more evident and should be paid enough attention.

**Table 3:** The mediation effects of relating factors between the three determinants and prehospital decision time (*N* = 1,032).

	**Job burnout**			**Age**			**Worrying about expenditure**		
	**Estimate**	**Est./S.E**.	***p*-Value**	**Estimate**	**Est./S.E**.	***p*-Value**	**Estimate**	**Est./S.E**.	***p*-Value**
**Multiple mediation model**									
**Total effect**	0.104	2.905	**0.004**	0.077	2.307	**0.021**	0.086	2.389	**0.017**
**Total indirect effect**	0.026	1.634	0.102	−0.029	−2.026	0.043	0.006	0.946	0.344
Specific indirect 1: social support	0.027	2.221	**0.026**	−0.039	−3.282	**0.001**	0.010	2.221	**0.026**
Specific indirect 2: medical related jobs	−0.005	−1.514	0.130	0.016	2.037	**0.042**	−0.007	−1.902	0.057
Specific indirect 3: type D personality	0.003	0.245	0.807	−0.005	−1.035	0.301	0.003	0.679	0.497
**Direct effect**	0.079	2.055	**0.040**	0.106	2.982	**0.003**	0.080	2.217	**0.027**

*Bold values indicate statistically signficant*.

Further analyses tried a simple mediation model to estimate the effect of social support and to avoid the interference of complex interactions (see Supplementary Figure 1). The partial mediating effect of social support accounted for 24.0% of the total effect for job burnout, and 11.6% of the total effect for worrying about expenditure.

## Discussion

In this study of 1,032 participants, job burnout, older age, worrying about possible expenditure, type D personality, engaging in medical-related jobs, and lack of social support were all found correlated with prehospital decision time delay. However, only the first three were retained in the multivariate analyses, proving themselves to be the most significant related factors. Social support, though not remarkably associated with decision time, partially mediated the relationship between job burnout, worrying about expenditure with prehospital decision time. These findings confirm the correctness of our hypotheses that burnout postpones the prehospital decision time and social support buffers the relationship between burnout and prehospital decision delay.

For the majority, working is always a crucial part of life. The experience at work closely relates to the mood and to some extent also reshapes our character (Dai, 2002). As the outcomes of excessive chronic stress at work, job burnout can lead to psychological disturbance such as insomnia, depressive symptoms, and use of psychotropic medications (Salvagioni et al., 2017) and be viewed as the predictors for worse prognosis in some somatic diseases. It is a widespread phenomenon in the modern world and has been regarded as a profound concern, which has caused enormous financial and emotional costs for companies and individual employees (Bakaç et al., 2021).

In this study, we tried to connect this “common” phenomenon with the behavior of seeking medical help and revealed a significant relationship between job burnout and prehospital decision delay. To the best of our knowledge, this is the first study that explores the association between these two things. Given the benefits of seeking medical care timely, finding the factors that help to promote early detection, early diagnosis and early treatment are of great importance. However, previous studies have paid more attention to acute diseases, which manifest as more serious symptoms, and found that some unmodifiable factors influence the prehospital delay (Cao et al., 2010; Mohan et al., 2018; Park et al., 2020). For most chronic diseases and some acute diseases at the initial stage, the manifestations are often mild and tolerable, and the timing to choose to go to the hospital varies significantly between individuals. As a result of this, it is possible that psychosocial factors have played a vital role in the formation of motivation for seeking medical examinations. Therefore, to enroll participants with previously tolerable body discomfort without restricting to a certain somatic disease was a drawback, perhaps also a strength of this research in finding the influence of psychosocial factors.

This conclusion can be corroborated by the finding that job burnout can result in the exhaustion of energy, make employees delay decisions (Roster and Ferrari, 2020), and be prone to have a more risky decision-making style (Michailidis and Banks, 2016). In addition, it is reported that burnout is a non-negligible cause and source of depression (Johansson et al., 2011); meanwhile, depression in patients with cardiac diseases is found associated with delay in seeking medical care (Arrebola-Moreno et al., 2020a). In fact, job burnout is often understood as the chronic depletion of an individuals' physical, emotional, and cognitive energy resources (Nguyen et al., 2010b; Salpigktidis et al., 2016). People with high levels of job burnout were more likely to experience conflicts inside and outside of work, which in turn would cause depression, anxiety, and other negative emotions and cognitive bias (Salvagioni et al., 2017).

There is also an interesting finding that although as high as 50.7% of respondents would endure the discomfort and 40.3% believed that delay was attributed to their business at work, statistical analyses demonstrated even a decrease in prehospital decision time as compared to the rest participants. Analogously, worrying about the possible expenditure was a determinant for prehospital decision delay; however, the actual income level did not correlate with the delay. It is probably not the business of work, but how the mind thought that really matters. The crucial part of the process in seeking medical help maybe not lie in how big objective obstacles are, but the lack of motivation inside.

Social support is considered to promote biological or behavioral adaptations under conditions of stress. Low social support may lead to mood disturbance (Åhlin et al., 2018), worse treatment compliance (Sousa et al., 2019), and unhealthy behaviors, which usually have a negative impact on the overall physical condition (Wang et al., 2017). In accord with previous studies, lack of social support was found as an important element for prehospital delay (Reisinger et al., 2018). Besides the direct correlation, this study also revealed that social support partially mediated the relationships between job fatigue and prehospital decision time. In other words, the delayed care-seeking manner caused by job burnout was partially attributed to the lack of social support. This is in line with the findings that enhancing social support can mitigate the extent of job burnout (Diehl et al., 2021; Yamoah, 2021). It also points out a direction that strengthening the social support may help reduce prehospital decision time and to facilitate disease prevention.

Consistent with previous studies (Zhang et al., 2016), this study also observed that aging was one of the determinants leading to the extension of prehospital decision time. The gradually decreased motor ability and lack of care and support might be an important reason. Engaging in medical-related jobs was found to be related to shortened decision time, which has also been reported by several prior studies (Nguyen et al., 2010a; Li et al., 2019). It is, however, impossible for the general public to master the knowledge of all the kinds of diseases and to self-diagnose. One recent review concludes that so far there is limited evidence for a relationship between prehospital delay and knowledge of symptoms (Mooney et al., 2012). The correlation between type D personality especially for negative affectivity and prehospital decision delay was another finding of this research, although this phenomenon has already been reported before (Zhang et al., 2020). In our study, a mediating effect of type D personality in the relationship between job burnout and the prehospital delay was not found, indicating that certain personalities might have exerted a non-negligible effect in the delay independent of job fatigue.

Unlike previous studies, this study has focused on the influence of psychosocial factors such as job burnout, social support, and type D personality, which we believe have played a non-negligible role and have not been paid enough attention to by the medical practitioner and policymakers. More importantly, these factors are a modifiable and perhaps essential supplement to the current disease prevention system. Under the circumstance that the graded diagnosis and treatment system in China has not been fully established and annual physical examination is still not a mainstream trend, realizing the significant impact of psychosocial factors may bring about a great improvement in promoting health. Future researches are still needed in figuring out the exact influence of burnout caused by prehospital delay on prognosis and finding measures to alleviate the negative impact of job fatigue. A more stringent design to minimize the recall bias in larger and restricted populations is also necessary.

There are also several limitations to this study. First of all, as for representing the general population in China, the sample size is still comparatively small. Given the difference in public health systems between countries, which significantly affects medical-seeking behavior, the generalization of the results worldwide needs to be further verified. Second, the data were collected during coronavirus disease 2019 (COVID-19) pandemic. Whether the conclusion drawn from this study can be extended needs to be treated with caution, although only 1 confirmed case of domestic infection has been reported nationwide during the period of data collection. Third, the propagation of questionnaires on the Internet may cause a bias toward younger participants with better living conditions. However, with the widespread use of smartphones in China, the proportion of participants aged over 40 in this study reached 44.4%. At last, the prehospital decision time, acquired based on the memories of participants can bring about recall bias. The reassuring thing is that the length of prehospital delay and self-assessment about the timeliness in seeking help are extremely consistent (*p* < 0.001), and the conclusions drawn in this study can be strongly supported by previous researches.

## Conclusion

In an internet-based survey, job burnout was found associated with prehospital decision delay. Social support partially mediated the relationship between burnout, worrying about expenditure with prehospital decision time. Job burnout and lack of social support, as two elements are commonly seen in the modern world, should be given enough consideration in disease prevention and treatment. Future researches are still needed to find out more effective intervention measures for burnout and to testify the conclusions in larger populations.

## Data Availability Statement

The raw data supporting the conclusions of this article will be made available by the authors, without undue reservation.

## Ethics Statement

The studies involving human participants were reviewed and approved by Medical Ethics Committee of Guangdong Provincial People's Hospital. Written informed consent to participate in this study was provided by the participants.

## Author Contributions

HY and CJ designed this study. HY, XS, and YC wrote the first draft. CJ, XY, YW, and WL did statistical analyses. QG revised the manuscript. HM and QG were senior physicians principally responsible for this study. All authors read and approved the final version of the manuscript.

## Funding

This study was funded by the High-level Hospital Construction Project of Guangdong Provincial People's Hospital (DFJH201811 and DFJH201922) and the National Key R&D Program of China (2018YFC2001805).

## Conflict of Interest

The authors declare that the research was conducted in the absence of any commercial or financial relationships that could be construed as a potential conflict of interest.

## Publisher's Note

All claims expressed in this article are solely those of the authors and do not necessarily represent those of their affiliated organizations, or those of the publisher, the editors and the reviewers. Any product that may be evaluated in this article, or claim that may be made by its manufacturer, is not guaranteed or endorsed by the publisher.
