# Supplementary material for: Job Burnout Is Associated With Prehospital Decision Delay: An Internet-Based Survey in China
Source: Front Psychol. 2022 Apr 11;13:762406. doi: 10.3389/fpsyg.2022.762406 (PMC9045849; doi:10.3389/fpsyg.2022.762406)
Supplement: Supplementary file 1 [file Data_Sheet_1.PDF]

Supplementary File 1 ----- Questionnaire

(translated from Chinese into English)

Investigation of the Psychological Factors on Prehospital Decision Delay

Dear Mr./ Ms,
Job burnout, certain personality, social support and other factors may cause delayed diagnose and treatment， resulting in irreparable consequences. This questionnaire is designed to explore their internal relationship, the results of which will be conducive to the improvement of disease prevention system in the future. Please feel free to fill in truthfully, thank you very much for your support and help.

This survey, including 50 choice questions， takes about 10 minutes to complete and participants remain anonymous.

1. Gender. [single choice]*

○ Male.

○ Female.

2. Your age. [single choice]*

○10-19 years.

○20-29 years.

○30-39 years.

○40-49 years.

○50-59 years.

○60-69 years.

○70-79 years.

○80 and over.

3. Occupation. [fill in the blanks]*

4. Educational background.[single choice]*

○ Primary Schools and Below.

○ Junior Middle School.

○ High school.

○ College (including undergraduate).

○ Graduate students and above.

5. Your monthly income. [single choice]*

○ No income.

○ $1-3000.

○ $3001-6000.

○ $6000-10000.

○ $10000-20000.

○ $20000 or more.

6. Whether you are engaged in medical related industry? [single choice]*

○ Yes.

○ No.

7. Your medical check-up frequency. [single choice]*

○ Hardly ever had a medical check-up.

○ Every ten years.

○ Every few years.

○ Once a year.

○ Several times per year.

8. When certain perceptible but tolerable symptom first occurs to you, your first choice is.[single choice]*

○ Endure for a while to see if the symptoms disappear.

○ Search related disorders online.

○ Contact relatives or friends in the medical field by WeChat, telephone etc.

○ Immediate access to nearby clinics.

○ Immediate access to major hospitals.

○ Can make a self-diagnosis.

9. When certain perceptible but tolerable symptom first occurs to you, how long does this discomfort take you, ideally, to go to the hospital for an examination? [single choice]*

○ Immediately.

○ 1-3 days.

○ 4-7 days.

○ 8-15 days.

○ 16-30 days.

○ 31-90 days.

○ 91-180 days.

10. Have you ever had the above conditions in the past and prompt access to a doctor as you think? [single choice]*

○ Every time.

○ Most of the time.

○ Part of the time.

○ Occasionally.

○ Hardly ever.

11. Have you ever have the above conditions in the past, and how much has your actual time to see a doctor been delayed on average compared with your ideal time to see a doctor?

[Enter numbers 0(0 days) to 180(180 days)]*

________________________________.

12. What are the factors that affect your prompt access to a doctor?[multiple choice]*

□ Work factors: too busy to ask for leave.

□ Work factor: tired of work and want to rest when free.

□ Personal reason: endure until self-healing.

□ Personal cause: fear of serious illness diagnosis.

□ personal reason: lack of motivation, want to be accompanied.

□ Economic reasons: worry about spending too much money.

□ Family reason: fear others would know, fear family would worry.

□ Family factors: need to look after family.

□ Objective reason: seeing a doctor is too troublesome and the process is complicated.

□ Objective reason: no clinic/hospital nearby.

□ Objective reasons: poor level of nearby hospitals.

□ Other.

13. Other factors. [blanks]

____

14. If you are not prompt for seeking medical treatment, will your worries about the health problems affect your normal life and work? [single choice]*

○ Not at all.

○ Slightly affect.

○ Moderately affect, but not easily perceived by others.

○ Seriously affect, easily perceived by others.

○ Can hardly live or work properly.

15. Your usual exercise frequency is: [single choice]*

○ Hardly exercise.

○ Not fixed, exercise every few months on average.

○ Exercise 1-3 times a month.

○ Exercise 1-2 times a week.

○ Exercise 3 times a week or more.

16.Your usual exercise intensity is: [single choice]*

○ Extremely mild , with slightly aggravated breath(e.g. strolling).

○ Mild, obviously able to feel breathing (e.g. walking quickly).

○ Moderate, with shortness of breath (e.g. running faster).

○ Vigorous, gasping, barely able to talk.

○ Extremely Vigorous, difficult to breath, unable to speak.

The following assessment concerning job burnout.

17. I feel emotionally drained from my work. [single choice]*

○ Never.

○ A few times a year or less.

○ Once a month or less.

○ A few times a month.

○ Once a week.

○ A few times a week.

○ Every day.

18. I feel used up at the end of the work day. [single choice]*

○ Never.

○ A few times a year or less.

○ Once a month or less.

○ A few times a month.

○ Once a week.

○ A few times a week.

○ Every day.

19. I feel fatigued when I get up in the morning and have to face another day on the job. [single choice]*

○ Never.

○ A few times a year or less.

○ Once a month or less.

○ A few times a month.

○ Once a week.

○ A few times a week.

○ Every day.

20. Working all day is really a strain for me. [single choice]*

○ Never.

○ A few times a year or less.

○ Once a month or less.

○ A few times a month.

○ Once a week.

○ A few times a week.

○ Every day.

21. I feel burned out from my work. [single choice]*

○ Never.

○ A few times a year or less.

○ Once a month or less.

○ A few times a month.

○ Once a week.

○ A few times a week.

○ Every day.

22. I've become more callous toward this job, Since I took it. [single choice]*

○ Never.

○ A few times a year or less.

○ Once a month or less.

○ A few times a month.

○ Once a week.

○ A few times a week.

○ Every day.

23.I am not as enthusiastic about my work as before. [single choice]*

○ Never.

○ A few times a year or less.

○ Once a month or less.

○ A few times a month.

○ Once a week.

○ A few times a week.

○ Every day.

24. I doubt the significance of my work. [single choice]*

○ Never.

○ A few times a year or less.

○ Once a month or less.

○ A few times a month.

○ Once a week.

○ A few times a week.

○ Every day.

25.I’ve become more unconcerned about my contribution to my work. [single choice]*

○ Never.

○ A few times a year or less.

○ Once a month or less.

○ A few times a month.

○ Once a week.

○ A few times a week.

○ Every day.

26. I deal very effectively with the problems in my work. [single choice]*

○ Never .

○ A few times a year or less.

○ Once a month or less.

○ A few times a month.

○ Once a week.

○ A few times a week.

○ Every day.

27. I’ve been making great contribution to the work. [single choice]*

○ Never.

○ A few times a year or less.

○ Once a month or less.

○ A few times a month.

○ Once a week.

○ A few times a week.

○ Every day.

28. In my opinion, I am good at my work. [single choice]*

○ Never.

○ A few times a year or less.

○ Once a month or less.

○ A few times a month.

○ Once a week.

○ A few times a week.

○ Every day.

29. I feel very happy when I finish some of my work. [single choice]*

○ Never.

○ A few times a year or less.

○ Once a month or less.

○ A few times a month.

○ Once a week.

○ A few times a week.

○ Every day.

30. I have accomplished many worthwhile things in this job. [single choice]*

○ Never.

○ A few times a year or less.

○ Once a month or less.

○ A few times a month.

○ Once a week.

○ A few times a week.

○ Every day.

31. I am confident that I can perform the job effectively. [single choice]*

○ Never.

○ A few times a year or less.

○ Once a month or less.

○ A few times a month.

○ Once a week.

○ A few times a week.

○ Every day.

Assessment of personality traits.

32. I make contact easily when I meet people. [single choice]*

○ False.

○ Rather false.

○ Neutral.

○ Rather true.

○ True.

33. I often make a fuss about unimportant things. [single choice]*

○ False.

○ Rather false.

○ Neutral.

○ Rather true.

○ True.

34. I often talk to strangers. [single choice]*

○ False.

○ Rather false.

○ Neutral.

○ Rather true.

○ True.

35. I often feel unhappy. [single choice]*

○ False.

○ Rather false.

○ Neutral.

○ Rather true.

○ True.

36. I am often irritated. [single choice]*

○ False.

○ Rather false.

○ Neutral.

○ Rather true.

○ True.

37. I often feel inhibited in social interactions. [single choice]*

○ False.

○ Rather false.

○ Neutral.

○ Rather true.

○ True.

38. I take a gloomy view of things. [single choice]*

○ False.

○ Rather false.

○ Neutral.

○ Rather true.

○ True.

39. I find it hard to start a conversation. [single choice]*

○ False.

○ Rather false.

○ Neutral.

○ Rather true.

○ True.

40. I am often in a bad mood. [single choice]*

○ False.

○ Rather false.

○ Neutral.

○ Rather true.

○ True.

41. I am a closed kind of person. [single choice]*

○ False.

○ Rather false.

○ Neutral.

○ Rather true.

○ True.

42. I would rather keep people at a distance. [single choice]*

○ False.

○ Rather false.

○ Neutral.

○ Rather true.

○ True.

43. I often find myself worrying about something. [single choice]*

○ False.

○ Rather false.

○ Neutral.

○ Rather true.

○ True.

44. I am often down in the dumps. [single choice]*

○ False.

○ Rather false.

○ Neutral.

○ Rather true.

○ True.

45. When socializing, I don't find the right things to talk about. [single choice]*

○ False.

○ Rather false.

○ Neutral.

○ Rather true.

○ True.

Social support is assessed below.

46. How many close friends whom you can turn to for support and help ? [single choice]*

○ None.

○1-2.

○3-5.

○6 or more.

47. Within nearly a year you’ve been :[single choice]*

○ Staying away from family and living alone.

○ Living with strangers most of the time, with changing accommodation frequently.

○ living with classmates, colleagues or friends.

○ living with family.

48. You and your neighbor :[single choice]*

○ Never cared about each other, and barely know each other.

○ May exhibit concern when encountering difficulties.

○ Some neighbors greatly care about you.

○ Most neighbors greatly care about you.

49. You and colleagues :[single choice]*

○ Never cared about each other, and barely know each other.

○ May exhibit concern when encountering difficulties.

○ Some colleagues greatly care about you.

○ Most colleagues greatly care about you.

50. Support and care received from family members. ("√" in the verified box)[matrix questions]*

	No	Few	General	Full support
A. couples (lovers).	○	○	○	○
B. Parents.	○	○	○	○
C. Children.	○	○	○	○
D. Siblings.	○	○	○	○
E.other members (e.g. sister-in-law).	○	○	○	○

51. In the past, financial and practical problem-solving support in an emergency came from :[multiple choice]*

□(1) No source.

□(2) The following sources (optional).

□A. Spouse.

□B. Other family members.

□C. Relatives.

□D. Colleagues.

□E. Work units.

□F. Official or semi-official organizations, such as party groups and trade unions.

□G. Religious and social organizations.

□H. Other. __________________

52. In the past, when you were in a difficult situation, those received comforts and concerns came from :[multiple topics]*

□(1) No source.

□(2) The following sources (optional).

□A. Spouse.

□B. Other family members.

□C. Relatives.

□D. Colleagues.

□E. Work units.

□F. Official or semi-official organizations, such as party groups and trade unions.

□G. Religious and social organizations.

□H. Other. _________________

53. How do you pour out when you are upset :[single choice]*

○ Never talk to anyone.

○ Only turn to one or two people.

○ When a friend asks you.

○ Take the initiative to pour out for support and understanding.

54. How do you ask for help when you are in trouble :[single choice]*

○ Don't accept help from others.

○ Seldom ask for help.

○ Sometimes ask for help.

○ Often ask for help from family, relatives and organizations when encountering difficulty.

55. For the organizing activities of groups (such as party organizations, religious organizations, trade unions, student unions, etc.), you :[single choice]*

○ Never participate.

○ Occasionally participate.

○ Regularly participate.

- Actively participate.
