# Supplementary material for: Job Burnout Is Associated With Prehospital Decision Delay: An Internet-Based Survey in China
Source: Front Psychol. 2022 Apr 11;13:762406. doi: 10.3389/fpsyg.2022.762406 (PMC9045849; doi:10.3389/fpsyg.2022.762406)
Supplement: Supplementary file 2 [file Table_1.DOCX]

Supplementary Material

# Supplementary Figure 1


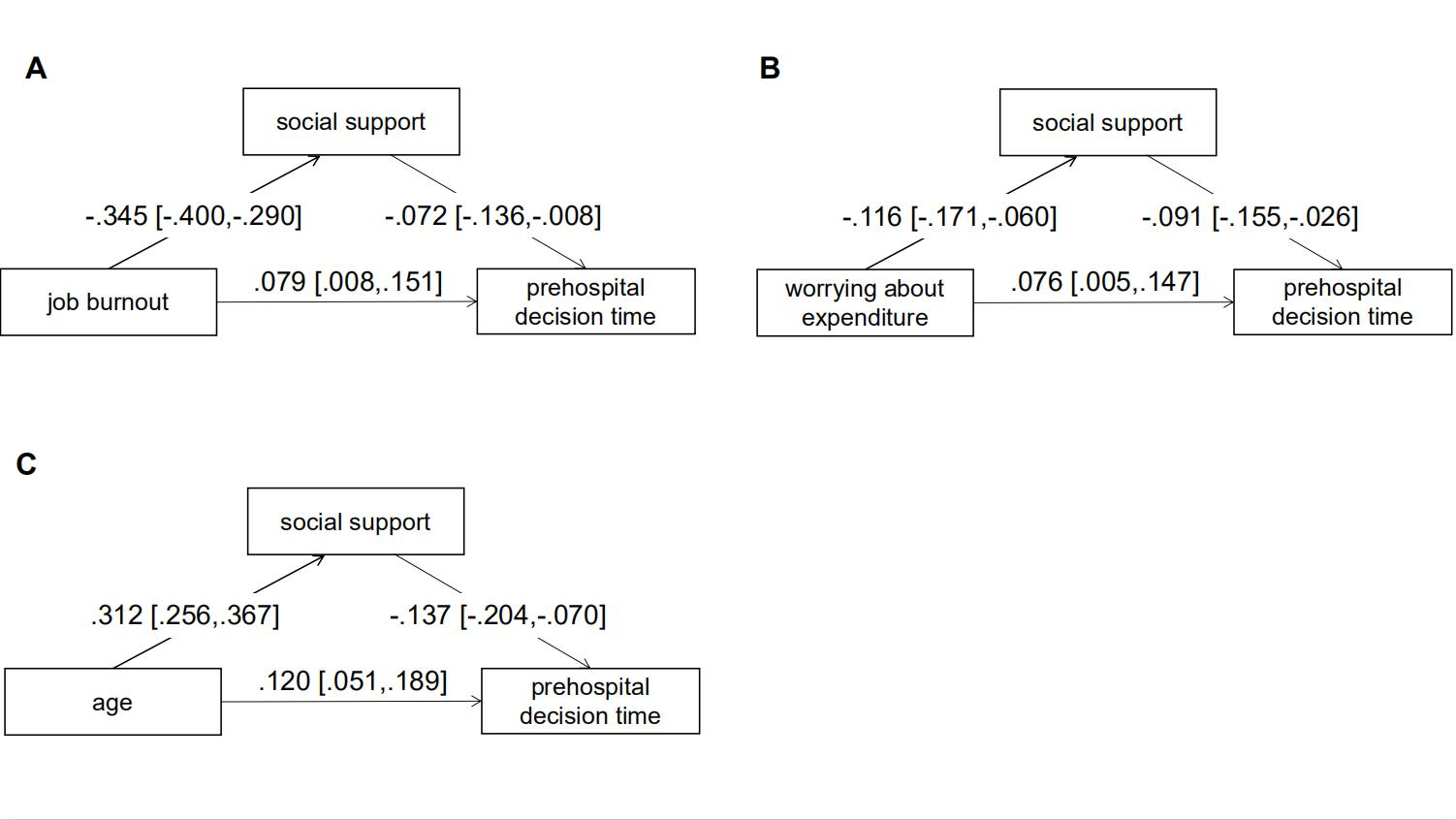


**Supplementary Figure 1.** Mediating effect of social support on the relationships between (A) job burnout, (B) worrying about expenditure, (C) age and prehospital decision time.

Note: Standardized model results are presented with covarience between independent variables and 95% confidential intervel by using bias-corrected percentile Bootstrap methods (iteration=10000 times)
